# Ultrasonography and clinicopathological features of breast cancer in predicting axillary lymph node metastases

**DOI:** 10.1186/s12885-022-10240-z

**Published:** 2022-11-09

**Authors:** Jiajia Xiong, Wei Zuo, Yu Wu, Xiuhua Wang, Wenqu Li, Qiaodan Wang, Hui Zhou, Mingxing Xie, Xiaojuan Qin

**Affiliations:** 1 Department of Ultrasound, Union Hospital, Tongji Medical College, Huazhong University of Science and Technology, 1277 Jiefang Avenue, Wuhan, 430022 China; 2 Hubei Province Key Laboratory of Molecular Imaging, Wuhan, 430022 China; 3 Department of Orthopedics, Wuhan Fourth Hospital, Wuhan, 430033 China

**Keywords:** Breast cancer (BC), Ultrasonography (US), Axillary lymph node metastasis (ALNM), Nomogram

## Abstract

**Background:**

Early identification of axillary lymph node metastasis (ALNM) in breast cancer (BC) is still a clinical difficulty. There is still no good method to replace sentinel lymph node biopsy (SLNB). The purpose of our study was to develop and validate a nomogram to predict the probability of ALNM preoperatively based on ultrasonography (US) and clinicopathological features of primary tumors.

**Methods:**

From September 2019 to April 2022, the preoperative US) and clinicopathological data of 1076 T1-T2 BC patients underwent surgical treatment were collected. Patients were divided into a training set (875 patients from September 2019 to October 2021) and a validation set (201 patients from November 2021 to April 2022). Patients were divided into positive and negative axillary lymph node (ALN) group according pathology of axillary surgery. Compared the US and clinicopathological features between the two groups. The risk factors for ALNM were determined using multivariate logistic regression analysis, and a nomogram was constructed. AUC and calibration were used to assess its performance.

**Results:**

By univariate and multivariate logistic regression analysis, age (*p* = 0.009), histologic grades (*p* = 0.000), molecular subtypes (*p* = 0.000), tumor location (*p* = 0.000), maximum diameter (*p* = 0.000), spiculated margin (*p* = 0.000) and distance from the skin (*p* = 0.000) were independent risk factors of ALNM. Then a nomogram was developed. The model was good discriminating with an AUC of 0.705 and 0.745 for the training and validation set, respectively. And the calibration curves demonstrated high agreement. However, in further predicting a heavy nodal disease burden (> 2 nodes), none of the variables were significant.

**Conclusion:**

This nomogram based on the US and clinicopathological data can predict the presence of ALNM good in T1-T2 BC patients. But it cannot effectively predict a heavy nodal disease burden (> 2 nodes).

## Introduction

Breast cancer (BC) is the most common cancer in women and the second most common cause of death from cancer among women in the world [1]. Axillary lymph node (ALN) status is an important prognostic factor for early BC [2, 3]. In current clinical practice, sentinel lymph node biopsy (SLNB) was accepted as the standard procedure to determine the axillary lymph node metastasis (ALNM) in BC at early stage. However, it was an invasive procedure [3], and intraoperative assessment of SLNs denies patients the opportunity to contribute to their treatment planning. Patients with clinical node negative disease (cN0) and one or two positive SLNs can be safely treated with breast-conserving surgery and radiotherapy [4]. Therefore, if the risk of ALN status can be predicted by a non-invasive method before surgery, unnecessary axillary surgery in patients with cN0 and one or two positive SLNs can be avoided.

At present, imaging examination is used as non-invasive method to confirm the ALN status in preoperative, such as Mammography (MG), Ultrasonography (US), Positron Emission Tomography-Computed Tomography (PET-CT) and Magnetic Resonance Imaging (MRI). Among the various imaging techniques, US is the primary method to preoperative evaluate the axilla in women with newly diagnosed BC, because it is economical, simple and widely used [5], and many studies show that the ALN morphological characteristics detected by axillary US were helpful for predicting ALNM [5, 6]. But the low sensitivity has limited the method for wide use. Because, for most patients with clinically node-negative early BC, axillary US has no positive signs, it can lead to false-negative results in patients with early-stage ALNM [7, 8]. Therefore, new methods not based on axillary examination are urgently needed to be explored, especially for patients with clinical early BC.

Recently, based on the development of artificial intelligence, particularly deep learning, people have begun to shift their attention from axillary US to the US features of primary tumor in early-stage BC, and found that the features of the primary tumor are also of good value for the prediction of ALNM [9, 10]. Artificial intelligence can automatically make a quantitative assessment of complex medical image characteristics and achieve increased accuracy in diagnosis with higher efficiency, however, the clinical application is difficult for which requiring early training and high cost [10, 11]. In recent years, some studies had used the US features of primary tumor to predict ALNM, such as tumor size, shape and structural distortion, etc. [12, 13]. Although there were established nomograms using axillary US and clinicopathological factors to predict ALNM of breast cancer [6], but few studies have focused on the effects of ultrasonic characteristics of primary tumor. So, the purpose of this study was to explore the risk factors of ALNM about US and clinicopathological features of primary tumor, and to build a nomogram model to predict the probability of ALNM based on those factors.

## Materials and methods

### Patient selection

**Fig. 1:**
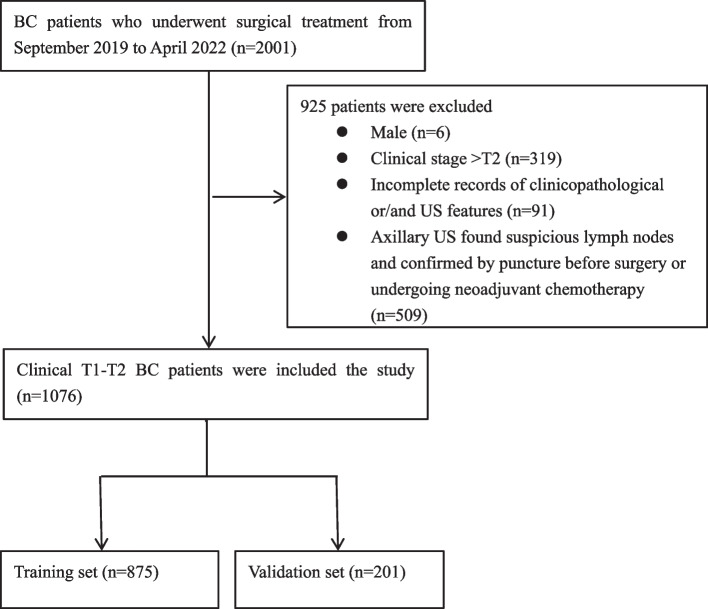
Flow chart of patient inclusion and exclusion criteria in the study. BC = breast cancer

Collected US and clinical features of 2001 BC patients who underwent surgical treatment in Cancer Center, Wuhan Union Hospital from September 2019 to April 2022. The inclusion criteria included female, clinical stageT1-T2, complete ultrasound and clinicopathological data. The excluded criteria were male, clinical stage greater than T2, incomplete record of clinical or ultrasound data, axillary US found suspicious lymph nodes and confirmed by puncture before surgery or undergoing neoadjuvant chemotherapy. Axillary US findings of suspicious lymph nodes include diffuse cortical thickness, asymmetric cortical thickness, and complete or near-complete absence of fatty hilum. Finally, a total of 1076 patients were included and 925 cases were excluded, the patient selection process was illustrated in Fig. 1. According to surgery time, patients were divided into a training set (875 patients from September 2019 to October 2021) and a validation set (201 patients from November 2021 to April 2022).

This retrospective study was conducted in accordance with the ethical standards of Declaration of Helsinki and was approved by the Ethics Committee of Tongji Medical College, Huazhong University of Science and Technology. The need for written informed consent to participate was waived by the Ethics Committee of Tongji Medical College, Huazhong University of Science and Technology due to retrospective nature of the study.

### US and clinical data source and collection

Patients' medical data were obtained from the medical records, all US images of the mass were digitally stored and retrospectively reviewed, three radiologists (with 6–10 years of experience in breast US) blinded to the axillary surgery results independently reviewed the US images. First, they independently analyze US images, when they encounter differences, they discuss together to reach a consensus. Only preoperative US performed before treatment is considered. US features of tumor included: multifocality or unifocal, maximum diameter of lesion, quadrant, shape, orientation, angular margin, spiculated margin and distance from the skin. If patients had multiple tumors on US, the largest tumor was selected for analysis. The clinicopathological features, including histologic types, histologic grades, hormonal receptors and molecular subtypes, were obtained by preoperative US-guided coarse needle biopsy (CNB).

### Pathological lymph node status

Lymph nodes metastasis was classified as macro-metastases(> 2 mm), micro-metastases (0.2-2 mm) or isolated tumor cells (ITC) (< 0.2 mm) based on pathologic examination. We described the nodal stage according to the TNM staging system from the eighth edition of the American Joint Committee on Cancer staging manual [14]. ITC were considered negative node [14]. Patients were divided into positive and negative groups based on ALND or SLNB results. For further analysis, patients with positive ALN were divided heavy nodal disease burden (> 2 nodes) group and low nodal disease burden (≤ 2 nodes) group based on axillary nodal disease burden.

### Molecular subtypes classification by IHC

The status of estrogen receptor(ER), progesterone receptor(PR) and human epidermal growth factor receptor 2(HER2) were was determined by immunohistochemical (IHC) staining. ER or PR was considered positive if there are at least 1% positive of the nuclear-staining cancer cells. Fluorescence in situ hybridization (FISH) was used to determine HER2 status in cases of IHC staining 2 + . According to the St Gallen International Expert Consensus 2013 [15], breast cancers are categorized into five subtypes as follows: luminal A (ER + and/or PR + , HER2 − , Ki67 < 14%), luminal B (ER + and/or PR + , HER2 − , Ki67 ≥ 14%), luminal B HER2 ( +) (ER + and/or PR + , HER2 + , any Ki67), HER2-enriched (ER − , PR − , HER2 +), and triple negative (TN) (ER − , PR − , HER2 −).

### Statistical analysis

Statistical analysis was performed using SPSS 22.0 and R Software ver.4.1.0. continuous data were presented as mean ± SD. Categorical variables were expressed as numbers and percentages of the group from which they were derived. Student’s *t*-test was used by continuous data. χ2 test and fisher exact tests were used to evaluate data from categorical variables.

Variables had statistically significant in the univariate analysis were used to fit the logistic regression model employing a ‘‘stepwise’’ variable selection procedure. Estimated parameters were reported as Odds Ratios with 95% confidence intervals and *p* values. The *p* < 0.05 was considered statistically significant. Post hoc analysis was conducted using the Bonferroni method to distinguish the differences among molecular subtypes and histologic grades.

The multiple logistic regression model was builted, and the representative nomogram of the model was constructed in the training set. The internal and external validation of our predictive model was assessed in both training set and validation set. For the validation, the calibration and discrimination were evaluated with Hosmer–Lemeshow goodness-of-fit test and the area under the curve (AUC). In the part of predicting a heavy nodal disease burden (> 2 nodes). χ2 test and fisher exact tests were used for data analysis, The *p* < 0.05 was considered statistically significant.

#### Results

### Patient characteristics

Of these 1076 patients, the mean age of selected patients was 52.0 ± 10.7 years (from 21 to 85 years), 510 (47.4%) of them had pathological positive axillary nodes (pN +), and other 566 (52.6%) of them had pathological negative axillary nodes (pN0). Among the 510 positive ALNs, patients with pN1, pN2 disease were 395(77.5%),115(22.5%)respectively. 221 (43.3%) of them had a heavy nodal disease burden (> 2 nodes), and other 289 (56.7%) of them had a low nodal disease burden (≤ 2 nodes). Among all patients, 355 (33.0%) patients received SLNB alone, 398 (37.0%) patients received ALND alone, and 323 (30.0%) patients received SLNB followed by ALND. Among the patients receiving SLNB alone, 15 patients with positive SLNB were omitted ALND, and 11 of them had one micro-metastasis; 2 of them had two micro-metastases; 2 of them had one macro-metastases. The comparison of the US and clinicopathological features of patients between our training set (*n* = 875) and validation set (*n* = 201) was listed in Table 1. The distribution of variables in the validation set was basically the same as that in the training set, with slight differences among the molecular subtypes (Table 1).

**Table 1:** Clinicopathologic and US features of the training and validation cohorts

Features	Training set (*n* = 875) N (%)	Validation set (*n* = 201) N (%)	*P* Value
Age(years)			0.430
≤ 50	410 (46.9%)	88 (43.8%)	
> 50	465 (53.1%)	113 (56.2%)	
Histologic types			0.676
Ductal	812 (92.8%)	185 (92.0%)	
Lobular	21 (2.4%)	7 (3.5%)	
Others	42 (4.8%)	9 (4.5%)	
Histologic grades			0.104
I	43 (4.9%)	5 (2.5%)	
II	393 (44.9%)	81 (40.3%)	
III	439 (50.2%)	115 (57.2%)	
Molecular subtypes			0.005
Luminal A	182 (20.8%)	33 (16.4%)	
Luminal B	374 (42.7%)	70 (34.8%)	
Luminal B HER2( +)	82 (9.4%)	19 (9.5%)	
HER2-enriched	129 (14.7%)	50 (24.9%)	
Triple negative	108 (12.3%)	29 (14.4%)	
Multifocality			0.096
Unifocal	724 (82.7%)	176 (87.6%)	
Multifocality	151 (17.3%)	25 (12.4%)	
Tumor location			0.436
Upper outer quadrant	518 (59.2%)	125 (62.2%)	
Others	357 (40.8%)	76 (37.8%)	
Maximum diameter			0.058
≤ 2 cm	328 (37.5%)	61 (30.3%)	
> 2 cm	547 (62.5%)	140 (69.7%)	
Shape			1.000*
Irregular	864 (98.7%)	199 (99.0%)	
Oval and round	11 (1.3%)	2 (1.0%)	
Orientation			0.270
Parallel	744 (85.0%)	177 (88.1%)	
Not parallel	131 (15.0%)	24 (11.9%)	
Angular margin			0.109
Yes	633 (72.3%)	134 (66.7%)	
No	242 (27.7%)	67 (33.3%)	
Spiculated margin			0.221
Yes	260 (29.7%)	51 (25.4%)	
No	615 (70.3%)	150 (74.6%)	
Distance from the skin			0.342
< 3 mm	174 (19.9%)	46 (22.9%)	
≥ 3 mm	701 (80.4%)	155 (77.1%)	

Note. *ALNM* Axillary lymph node metastasis, *HER2* Human epidermal growth factor receptor 2

^*^Fisher's exact test

### Univariate analysis of ALNM

Univariate analysis was conducted in the training set (Table 2). In histologic grades, compared with grade I, histological grade II or III were more prone to ALNM (all *P* < 0.05), but there is no statistically significant difference between grade II and III. In molecular subtypes, the ALNM rates were 72.0% for luminal B HER2 ( +), 51.9% for luminal B, 50.0% for TN, 48.1% for HER2-enriched, and 37.4% for luminal A. Luminal B HER2 ( +) was more prone to ALNM, with a statistically significant difference compared to all other types (all *p* < 0.05). There was a statistically significant difference between luminal B HER2 (-) and luminal A (*p* < 0.05). But there were no statistically significant difference between HER2-enriched and TN compared with luminal B HER2 (-) or luminal A (all *p* > 0.05).

**Table 2:** Clinicopathologic and US features of BC patients who presented with and without ALNM

Features	ALNM (*n* = 437) N (%)	Non-ALNM (*n* = 438) N (%)	*P* Value
Age(years)			0.009
≤ 50	224 (54.6%)	186 (45.4%)	
> 50	213 (45.8%)	252 (54.2%)	
Histologic types			0.017
Ductal	415 (51.1%)	397 (48.9%)	
Lobular	10 (47.6%)	11 (52.4%)	
Others	12 (28.6%)	30 (71.4%)	
Histologic grades			0.000
I	6 (14.0%)	37 (86.0%)	
II	199 (50.6%)	194 (49.4%)	
III	232 (52.8%)	207 (47.2%)	
Molecular subtypes			0.000
Luminal A	68 (37.4%)	114 (62.6%)	
Luminal B	194 (51.9%)	180 (48.1%)	
Luminal B HER2( +)	59 (72.0%)	23 (28.0%)	
HER2-enriched	62 (48.1%)	67 (51.9%)	
Triple negative	54 (50.0%)	54 (50.0%)	
Multifocality			0.024
Unifocal	349 (48.2%)	375 (51.8%)	
Multifocality	88 (58.3%)	63 (41.7%)	
Tumor location			0.000
Upper outer quadrant	294 (56.8%)	224 (43.2%)	
Others	143 (40.1%)	214 (59.9%)	
Maximum diameter			0.000
≤ 2 cm	131 (39.9%)	197 (60.1%)	
> 2 cm	306 (55.9%)	241 (44.1%)	
Shape			0.365
Irregular	433 (50.1%)	431 (49.9%)	
Oval and round	4 (36.4%)	7 (63.6%)	
Orientation			0.646
Parallel	374 (50.3%)	370 (49.7%)	
Not parallel	63 (48.1%)	68 (51.9%)	
Angular margin			0.665
Yes	319 (50.4%)	314 (49.6%)	
No	118 (48.8%)	124 (51.2%)	
Spiculated margin			0.000
Yes	164 (63.1%)	96 (36.9%)	
No	273 (44.4%)	342 (55.6%)	
Distance from the skin			0.000
< 3 mm	109 (62.6%)	65 (37.4%)	
≥ 3 mm	328 (46.8%)	373 (53.2%)	

Note. *BC* Breast cancer, *ALNM* Axillary lymph node metastasis, *HER2* Human epidermal growth factor receptor 2

### Multivariate analysis of ALNM

Combining US and clinicopathological features of the tumor, binary logistic regression analysis as shown in Table 3. The results revealed that age, molecular subtypes, histologic grades, tumor location in the upper outer quadrant, maximum diameter, spiculated margin and distance from the skin are independent predictors of ALNM.

**Table 3:** Multivariate logistic regression analysis for predicting ALNM

Features	β	*P* Value	OR(95%CI)
Age ≤ 50y vs > 50y	0.410	0.006	1.507 (1.127–2.016)
Molecular subtypes		0.005	
Luminal B vs Luminal A	0.358	0.100	1.431 (0.933–2.194)
Luminal B HER2( +) vs Luminal A	1.083	0.001	2.954 (1.579–5.524)
HER2-enriched vs Luminal A	0.161	0.560	1.175 (0.683–2.020)
Triple negative vs Luminal A	0.261	0.376	1.298 (0.729–2.313)
Histologic grades		0.005	
II vs I	1.555	0.001	4.737 (1.838–12.212)
III vs I	1.557	0.002	4.742 (1.785–12.603)
Upper outer quadrant vs others	0.691	0.000	1.995 (1.486–2.679)
Maximum diameter > 2 cm vs ≤ 2 cm	0.504	0.001	1.655 (1.221–2.242)
Spiculated margin (yes vs no)	0.789	0.000	2.200 (1.594–3.037)
Distance from the skin < 3 mm vs ≥ 3 mm	0.591	0.002	1.806 (1.247–2.615)
Constant	-3.828	0.000	0.022

Note. *ALNM* Axillary lymph node metastasis, *HER2* Human epidermal growth factor receptor 2

### Nomogram development and validation

**Fig. 2:**
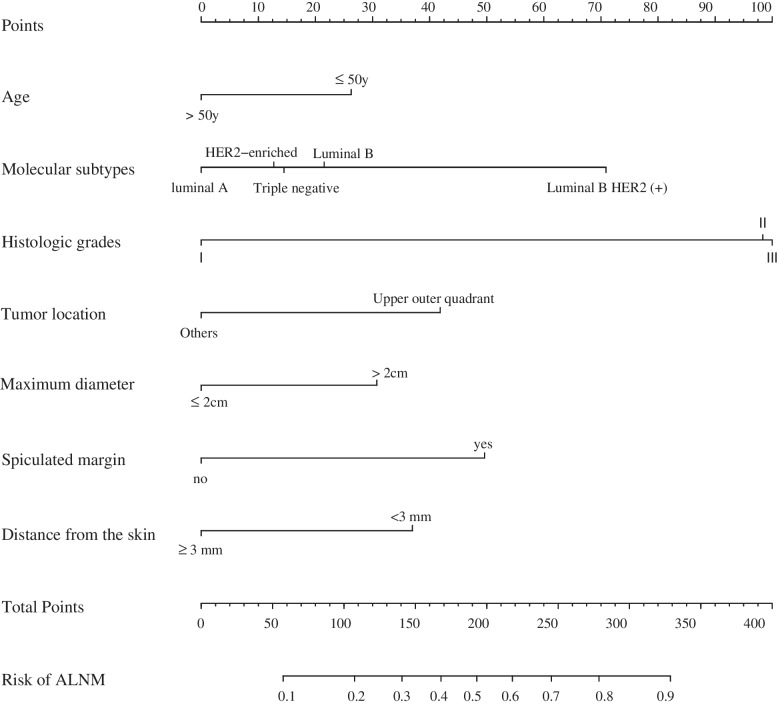
A nomogram for predicting ALNM. Age, molecular subtypes, histologic grades, tumor location, maximum diameter, spiculated margin and distance from the skin were finally selected to develop the model. ALNM = axillary lymph node metastasis

**Fig. 3:**
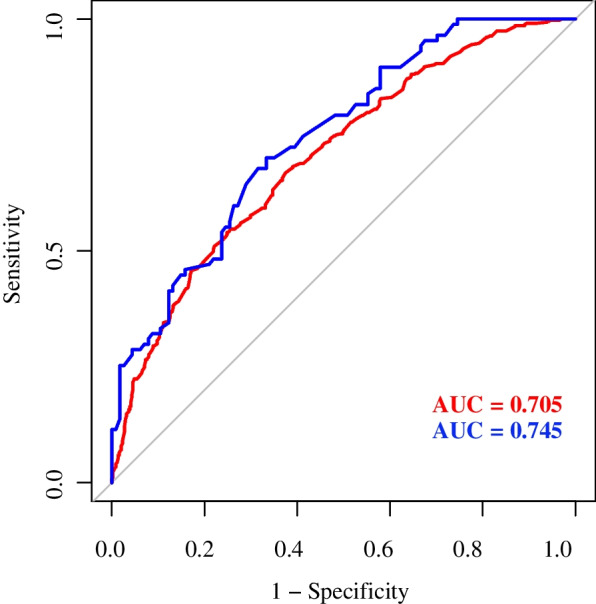
Performance of the nomogram were measured using the area under the receiver operating characteristic (ROC) curve, the AUC of the model was 0.705 (95% CI: 0.671–0.739) and 0.745 (95% CI: 0.679–0.812) for the training (red curve) and the validation (blue curve) set, respectively

**Fig. 4:**
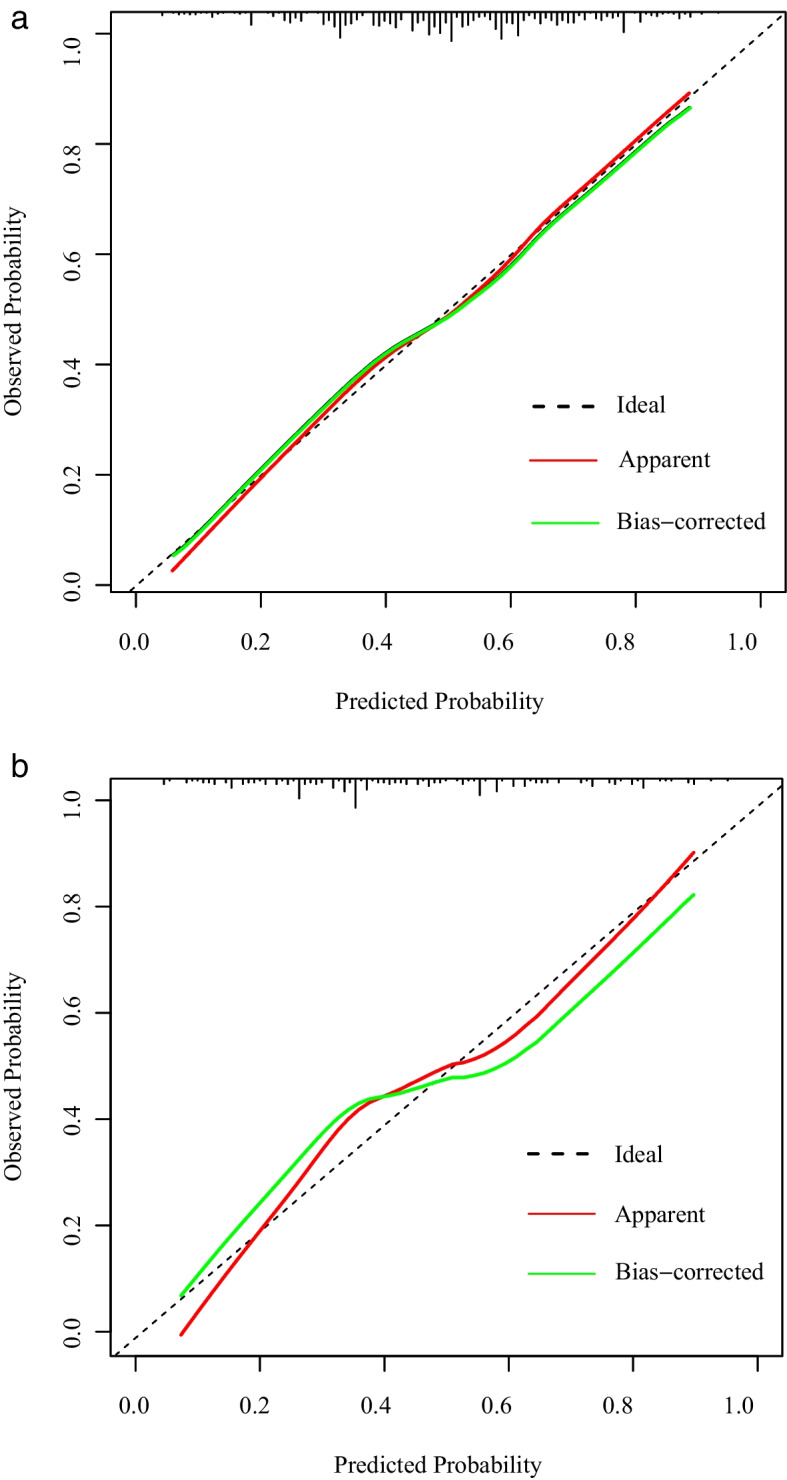
Calibration curve for predicting risk of ALNM in the training set (**a**) and validation set (**b**), Hosmer–Lemeshow goodness of fit test (all *p* > 0.05). ALNM = axillary lymph node metastasis

A nomogram to predict the likelihood of ALNM was developed based on the results of the multivariate logistic regression, points were assigned to each variable, then summed to yield the total number of points (Fig. 2). The model was good discriminating with an AUC of 0.705 (95% confidence interval [CI]: 0.671–0.739) and 0.745 (95% CI: 0.679–0.812) for the training and the validation set, respectively (Fig. 3). Moreover, the calibration plots presented excellent agreement in the training and the validation set (Figs 4a and b).

### *Predicting a heavy nodal disease burden (*> *2 nodes)*

Among the 437 positive ALN patients in the training set, 179 (41.0%) of them had a heavy nodal disease burden (> 2 nodes), and other 258 (59.0%) of them had a low nodal disease burden (≤ 2 nodes). Univariate analysis showed that, of all preoperative ultrasound and clinicopathological features, no variable was associated with a heavy nodal disease burden (> 2 nodes), (Table 4).

**Table 4:** Univariate analysis of clinicopathological and US features of primary tumors and axillary nodal disease burden in the training set

Features	Low nodal disease burden (≤ 2 nodes) N (%)	Heavy nodal disease burden (> 2 nodes) N (%)	*P* Value
Age(years)			0.355
≤ 50	137 (61.2%)	87 (38.8%)	
> 50	121 (56.8%)	92 (43.2%)	
Histologic types			0.115*
Ductal	244 (58.8%)	171 (41.2%)	
Lobular	4 (40.0%)	6 (60.0%)	
Others	10 (83.3%)	2 (16.7%)	
Histologic grades			0.929*
I	4 (66.7%)	2 (33.3%)	
II	119 (59.8%)	80 (40.2%)	
III	135 (58.2%)	97 (41.8%)	
Molecular subtypes			0.277
Luminal A	41 (60.3%)	27 (39.7%)	
Luminal B	112 (57.7%)	82 (42.3%)	
Luminal B HER2( +)	42 (71.2%)	17 (28.8%)	
HER2-enriched	35 (56.5%)	27 (43.5%)	
Triple negative	28 (51.9%)	26 (48.1%)	
Multifocality			0.991
Unifocal	206 (59.0%)	143 (41.0%)	
Multifocality	52 (59.1%)	36 (40.9%)	
Tumor location			0.594
Upper outer quadrant	171 (58.2%)	123 (41.8%)	
Others	87 (60.8%)	56 (39.2%)	
Maximum diameter(cm)			0.889
≤ 2	78 (59.5%)	53 (40.5%)	
> 2	180 (58.8%)	126 (41.2%)	
Shape			1.000*
Irregular	256 (59.1%)	177 (40.9%)	
Oval and round	2 (50.0%)	2 (50.0%)	
Orientation			0.066
Parallel	228 (60.8%)	147 (39.2%)	
Not parallel	30 (48.4%)	32 (51.6%)	
Angular margin			0.243
Yes	75 (63.6%)	43 (36.4%)	
No	183 (57.4%)	136 (42.6%)	
Spiculated margin			0.332
Yes	92 (56.1%)	72 (43.9%)	
No	166 (60.8%)	107 (39.2%)	
Distance from the skin			0.163
< 3 mm	57 (53.3%)	50 (46.7%)	
≥ 3 mm	201 (60.9%)	129 (39.1%)	

Note. *HER2* Human epidermal growth factor receptor 2

*Fisher's exact test

## Discussion

Lymph node metastasis in BC is not only the key factor determining the overall staging and prognosis, but also important for the choice of treatment modalities. Therefore, early understanding of ALN status becomes more important. Imaging examinations, include US and MRI, are the optional means of preoperative lymph node assessment and it is helpful for making optimal treatment decisions in clinical practice [3, 5]. However, the sensitivity was insufficient in early BC. So, more sensitive ways need to be developed.

In this study, age, molecular subtypes, histologic grades, upper outer quadrant, size, spiculated margin and distance from the skin were risk factors for ALNM. In order to better predict the possibility, we developed and validated a simple-to-use nomogram used these risk factors. It displayed a good performance with AUC of 0.705 (95% CI: 0.671–0.739) and 0.745 (95% CI: 0.679–0.812) for the training and the validation set, respectively. Two studies had also established nomograms to predict ALNM based on US and clinicopathological features [16, 17]. Both results also showed a good predictive value, the AUC was 0.731–0.848, and were slightly higher than this study. The reason was more risk factors, such as lymphovascular invasion (LVI), blood flow signal of the mass and ALN descriptors (shape, cortical thickness and long-to-short ratio) were included. LVI is closely related to ALNM and a powerful factor in predicting it. However, LVI is obtained after complete resection of the tumor, which has a certain lag. But in our study, all factors can be obtained before operation, the clinicopathological factors we selected can be obtained by ultrasound-guided hollow needle biopsy. So, this study allows to estimate the possibility of ALNM before surgery. Blood flow signal was easily affected by instruments and operators. So, we excluded this index when building the model. ALNs were not included in this study, one reason is that US is difficult to identify SLNs, there are often multiple ALNs in axillary and the morphology is diverse.

In the predictive model of this study, histological grades were the most important variable. Histological grade III showcased the highest predictive value, compared with patients with grade I, the OR is 4.742. In previous studies, histological grade was also demonstrated a significantly risk factors for ALNM [6, 12, 16]. Tumor with high histological grade means lower differentiation and higher malignancy, and is more prone to recurrence and metastasis. The reason may be the tumor with different histological grades shows distinct molecular profiles at the genomic, transcriptomic, and immunohistochemical levels, which are significantly associated with prognosis. Additionally, there may be an interaction between tumor size and histological grade through proliferation related genes [18].

Molecular subtypes were also an important factor in ALNM, but the controversy regarding the relationship between molecular subtypes and ALNM is existing. Jones et al. [19] found that breast cancer subtypes had no association with nodal positivity, N stage, or the absolute number of nodes involved among 453 patients who underwent breast-conserving surgery for stage I-II breast cancer. But more researchers thought that molecular subtypes were associated with positive ALNs [20–27]. However, the relationship between molecular subtypes and positive lymph node rate was also controversial. Some researchers had suggested that non-luminal subtypes (TN or HER2-enriched) tumors had higher incidence of ALNM than luminal subtypes [22, 23]. Other many researchers had reported that patients with non-luminal subtypes, including TN, have a lower incidence of lymph node metastases than those with luminal subtypes [24–27].

Our study showed that molecular type was associated with ALNM, and the nomogram showed it was the second important variable in the prediction model. The ALNM rate of patients with luminal B HER2 ( +) was the highest (72.0%), and significantly higher than other subtypes. This is consistent with some research [20–22, 25]. The reason may be that luminal B and HER2 positive have a high possibility of ALNM respectively. Synergy of luminal B and HER2 further promote the risk of ALNM. Luminal B (51.9%) was the second risk factor of ALNM. Previous studies had also shown that luminal type is more prone to ALNM than TN and HER2-enriched [26, 27]. but the reason is unclear. The risks of subtypes with TN, HER2-enriched and luminal A were lower than subtypes with Luminal B HER2( +). Although TN has a worse prognosis, some studies found it had a lower risk of ALNM [24–26]. So, the poor prognosis of TN breast cancers may be due to a higher propensity for distant (rather than regional) spread [23, 25]. Luminal A subtype has the best prognosis. Some studies also thought it has a lower risk of ALNM than other subtypes [22, 23]. This is similar to our results. The reason may be due to that luminal A subtype has low expression of Ki67 is which a well-established cell proliferation marker in cancer. Our study also showed that luminal A had the lowest risk of ALNM. HER2-enriched is a strong independent predictor of nodal metastasis in breast cancer, HER2-positive status was associated with an increased risk of ALNM at diagnosis compared to HER2-negative status [25]. However, there were no significant difference between TN and HER2-enrich or HER2-enrich and luminal A in this study.

In this study, spiculated margin was the third important variables in the prediction model. spiculated margin is one of the important characteristics of tumor invasive growth, and is more prone to ALNM. The reason may be the over expression of VEGF and MMP-9 in BC patients with burr sign [28]. VEGF can enhance angiogenesis and vascular and lymphatic permeability, and aids the proliferation and metastasis of tumor cells [29]. MMP-9 can dissolve in type-IV collagen, leading to damage to basement membranes, and has a very important role in the metastasis of cancer cells [30].

Location and size of primary tumor are also important indicator of prognosis [31, 32]. Some researchers [6, 16] found there was an increase in ALNM with increasing size of tumor. Our study also showed that tumors > 2 cm were more likely to have ALNM (OR = 1.655). The relationship between tumor size and ALNM is complex, it varies by molecular types, genetic background and expression of molecules that determine tumor growth and lymphatic metastasis may play an important role [33]. Tumors located in the upper outer quadrant increase incidence of lymph node positivity and are likely to have more number of ALNs [31]. Our study also showed tumors locate in the outer upper quadrant (OR = 1.995) were more likely to have ALNM. According to breast lymphatic drainage and tumor prevalence, BC was most common in the upper outer quadrant and was more likely to drain to the axilla [34].

Distance from the skin was found a risk factor of ALNM in our study. The natural dominant drainage for the outflow of lymph from the superficial areas of the breast is to the ALNs. This pathway plays a primary role in the initial stages of breast cancer. The superficial lymphatic drainage, as described by Sappey, was located from the skin to a 3 mm depth [35, 36]. Our study showed that tumors distance from the skin < 3 mm were more likely to have ALNM (OR = 1.806).

Age of onset also was associated with ALNM [37]. In our study. the mean age of diagnosis for breast cancer patients is 52.0 years old. Research has found when compared to the older patients, the younger patients present with several poor clinical indicators, including a stronger association with high-grade tumors, LVI, lymph-node involvement, more likely to be HER2-positive and triple-positive disease [37]. Our study also showed that less than 50 years old is an independent risk factor for ALNM (OR = 1.507).

Our study had several limitations. First, our study was a single-center retrospective study which was limited by the deficient collection of risk factors related to BC, such as menstruation and fertility. Second, there were subtle differences between training and validation set which may reduce the reliability of validation. Further research focused on validation will extend the generalized use of the nomogram. At last, studies had shown that elasticity was associated with ALN status [38], however, our study was a retrospective study and lacked data on elastography, more prospective studies may be needed in the future to further explore.

## Conclusions

In conclusion, the clinical approach to ALN management of BC patients is becoming more and more conservative. So, accurate assessment or/and prediction of ALN status before opreation are helpful to determine the best treatment plan. Our study developed and validated a prognostic nomogram with preoperative US and clinicopathological features, for the prediction of ALNM in patients with T1-T2 breast cancer. This nomogram performed well and might be helpful in risk stratification and decision-making for early BC patients. However, this study is poor prediction in a heavy nodal disease burden (> 2 nodes).

## Data Availability

The datasets used and/or analysed during the current study available from the corresponding author on reasonable request.

## Abbreviations

ALNM: Axillary lymph node metastasis
ALN: Axillary lymph node
AUC: Area under the curve
BC: Breast cancer
CI: Confidence interval
CNB: Coarse needle biopsy
ER: Estrogen receptor
HER2: Human epidermal growth factor receptor 2
LVI: Lymphovascular invasion
MG: Mammography
MRI: Magnetic Resonance Imaging
PET-CT: Positron Emission Tomography-Computed Tomography
PR: Progesterone receptor
ROC: Receiver operating characteristic
SLNB: Sentinel lymph node biopsies
SLN: Sentinel lymph node
TN: Triple negative
US: Ultrasonograghy
